# Assessment of Water Intake among Chinese Toddlers: The Report of a Survey

**DOI:** 10.3390/nu16132012

**Published:** 2024-06-25

**Authors:** Yiding Zhuang, Zhencheng Xie, Minghan Fu, Hongliang Luo, Yitong Li, Ye Ding, Zhixu Wang

**Affiliations:** 1 Department of Maternal, Child and Adolescent Health, School of Public Health, Nanjing Medical University, Nanjing 211166, China; zhuangyiding@stu.njmu.edu.cn (Y.Z.); zhenchengxie@njmu.edu.cn (Z.X.);; 2 Danone Open Science Research Center for Life-Transforming Nutrition, Shanghai 201204, China; 3 The Institute of Nutrition and Food Science, Nanjing Medical University, Nanjing 211166, China

**Keywords:** China, toddlers, total water intake, water from beverages, water from foods, adequate intake

## Abstract

Toddlerhood (aged 13~36 months) is a period of dietary transition, with water intake being significantly influenced by parental feeding patterns, cultural traditions, and the availability of beverages and food. Nevertheless, given the lack of applicable data, it is challenging to guide and evaluate the water intake of toddlers in China. In this study, our objectives were to assess the daily total water intake (TWI), evaluate the consumption patterns of various beverages and food sources contributing to the TWI, determine the conformity of participants to the adequate intake (AI) recommendation of water released by the Chinese Nutrition Society, and analyze the various contributors to the daily total energy intake (TEI). The data for the assessment of water and dietary intake were obtained from the cross-sectional dietary intake survey of infants and young children (DSIYC, 2018–2019). A total of 1360 eligible toddlers were recruited in the analysis. The differences in related variables between two age groups were compared by Mann–Whitney U test and Chi-Square test. The potential correlation between water and energy intake was examined utilizing age-adjusted partial correlation. Toddlers consumed a median daily TWI of 1079 mL, with 670 mL (62.3%, r = 0.752) derived from beverages and 393 mL (37.7%, r = 0.716) from foods. Plain water was the primary beverage source, contributing 300 mL (52.2%, r = 0.823), followed by milk and milk derivatives (MMDs) at 291 mL (45.6%, r = 0.595). Notably, only 28.4% of toddlers managed to reach the recommended AI value. Among these, toddlers obtain more water from beverages than from foods. The median daily TEI of toddlers was 762 kcal, including 272 kcal from beverages (36.4%, r = 0.534) and 492 kcal from foods (63.6%, r = 0.894). Among these, the median daily energy intake from MMDs was 260 kcal, making up 94.6% of the energy intake from beverages (r = 0.959). As the pioneer survey on TWI of toddlers in China based on nationally representative data, attention to the quality and quantity of water intake and actions to better guide parents by both individuals and authorities are eagerly anticipated. Additionally, the revision of the reference value of TWI for Chinese toddlers is urgently required.

## 1. Introduction

Toddlerhood (aged 13~36 months) is a crucial period during which toddlers gradually shift from a milk-based diet into a more diversified eating pattern. This transition is imperative for ensuring the intake of energy, water, and other nutrients, which are crucial for supporting the growth and development of toddlers. Furthermore, this transition contributes to the formation of healthy eating behaviors, which have a long-lasting and profound impact on their overall well-being [1,2].

Water makes up a significant portion of human tissue, accounting for 60~65% of adult body weight and 70~80% in toddlers [3]. Water also plays a crucial role in a variety of physiological processes, metabolic functions, and temperature balance [4]. Healthy adults have efficient mechanisms for regulating water balance in their bodies, whereas toddlers lack these mechanisms. Toddlerhood is typically one of the periods where children engage in increased physical activities, accompanied by more active sweat glands and higher water loss. However, their renal function remains immature, and their ability to perceive and express thirst is relatively weak. Furthermore, toddlers tend to be more susceptible to diarrhea, vomiting, and fever [5,6,7]. Consequently, all these factors put them at risk of dehydration.

The total water intake (TWI) comprises water obtained from both beverages and foods. Understanding the TWI of a certain population and its sources is crucial to ensure adequate water intake for that population. In recent years, many countries have developed recommended values for the TWI of residents by utilizing population water intake data. For toddlers, however, the deficiency of basic data on their water intake poses a challenge to the development of adequate intake (AI) value. For instance, for toddlers aged 13~23 months, the European Food Safety Authority established an AI value of 1100 to 1200 mL/day for TWI, determined through interpolation due to the unavailability of intake data [8]. The AI value of TWI for toddlers, as proposed by the Chinese Nutrition Society (CNS), is mathematically calculated from the sum of water provided by breast milk and complementary food. The CNS reported that the average water intake from breast milk was 480 mL/day, while water from complementary food was unknown. Thus, in accordance with the WHO recommendation of 550 kcal/d of energy for complementary foods and in consideration of recommended dietary allowance raised in the United Sates, which indicated that infants and children required 1.5 mL of water for every 1 kcal of energy consumed, the water intake from complementary food is extrapolated at 825 mL/d. Therefore, the AI value of TWI for toddlers in China is set as 1300 mL/d (480 mL from breast milk and 825 mL from complementary foods) [9].

As mentioned above, toddlerhood is a period of transformation in dietary patterns, so toddlers’ TWI is highly influenced by their parents’ feeding patterns, cultural customs, and accessibility to beverages and food, which vary between China and other countries. These differences among various countries may result in toddlers having different levels of TWI, with varying contributions from beverages and food to their TWI. Quite a few researchers have voiced their concerns about whether these recommendations, not grounded on actual survey data, were suitable in China [10,11]. Therefore, initiating a nationwide survey on the TWI of toddlers (aged 13~36 months) in China will serve as a pivotal step in developing recommended values for water intake for this population. Currently, as far as we know, there have been only a handful of surveys available on the water intake of children in China, and none of these surveys specifically targeted toddlers or considered the concept of TWI [11,12,13,14].

Therefore, based on the nationally representative sample in China, our primary objective was to assess the daily TWI and total energy intake (TEI) among toddlers of different ages, as well as the beverage-food consumption patterns and contributions to their TWI and TEI. The secondary aim was to conduct a comparative analysis between the TWI of toddlers and the AI value established by the CNS, and to assess the extent to which beverages and food sources contribute to the TWI in scenarios based on their compliance with the AI value [9]. The ultimate goal was to examine the correlation between the water and energy intake from different sources, the TWI and TEI, as well as water or energy derived from beverages and foods. We further hypothesized that the participants’ TWI did not meet the recommended AI value, and there was a strong correlation between TWI and the intake of plain water.

## 2. Materials and Methods

### 2.1. Study Sample

Our study utilized data from the 2018–2019 cross-sectional dietary intake survey on infants and young children in China (DSIYC). In view of the regional differences in geographic location, urban-rural disparities, income inequality, and annual birth rate, the provinces and municipalities covered were as follows: Beijing, Shanghai, Anhui, Fujian, Guangdong, Hebei, Henan, Hubei, Jiangsu, Liaoning, Sichuan, Yunnan, and Zhejiang, representing 13 out of 34 provincial administrative regions in China. Each region was subdivided into urban and rural areas, with a city established in each region to facilitate the survey. Subsequently, all the study samples were randomly recruited guided by the Maternal and Child Health Centre.

The inclusion criteria for children were: healthy children aged 13 to 36 months who were full-term singletons born at 37 to 42 weeks’ gestation, weighing 2500 to 4000 g. Children who were born preterm (gestational age < 37 weeks), post-term (gestational age ≥ 42 weeks), low birth weight (birth weight < 2500 g), or macrosomia (birth weight > 4000 g) were excluded. The parents/guardians of these children must have sufficient understanding, cooperation, and cognitive ability as well as question-and-answer capacity.

To determine the required sample size for the study, we used the formula $N=\frac{{\left(Z_{\alpha }\times S\right)}^{2}}{d^{2}}$ with a 95% confidence level (Z_α_ = 1.96), an estimated standard deviation of 1524 mL for TWI intake (S), and the maximum permissible error of 100 mL (d). This calculation suggested we needed at least 887 participants. Finally, water and dietary data of 1360 participants aged 13~36 months were collected, including 682 aged 13~24 months, and 678 aged 25~36 months [10,15].

### 2.2. Data Collection and Analysis

Well-trained investigators utilized an online diary, compatible with Phone/Tablet/iPad devices to assess the dietary intake of children. The online diary included a comprehensive food list with a food atlas to accurately estimate the water and dietary intake. Additional details have been presented in our previous report [16]. For children who were being breastfed, the frequency and duration of their mother’s daily breastfeeding were recorded, providing an estimate of their breast milk intake. Due to practical considerations, the online diaries were recorded two days (one weekday and one weekend) for breastfed children and four days (two weekdays and two weekends) for non-breastfed children.

The study was conducted in collaboration with Danone Open Science Research Centre for Life-Transforming Nutrition (Shanghai, China). Data collection was outsourced to a third-party organization, Taylor Nelson Sofres (Shanghai, China). Prior to the formal survey, a pilot survey was conducted to testify the feasibility and scientific validity of dietary data collection. All investigators must undergo training in survey techniques prior to conducting the study. In order to ensure authenticity of the data submitted by the participants and to prevent duplication, investigators were available to guide participants to upload their dietary data, thus maintaining a high level of participation. The raw data retrieved from the original recordings were promptly reviewed and scrutinized for the completeness and validity of the results.

Beverages are categorized into the following five main groups: plain water, milk and milk derivatives (MMDs), fruit and vegetables juices (FVJs), sugar-sweetened drinks (SSDs), and plant-based protein drinks (PBDs) (Table 1). Foods are categorized into the following five main groups: staple food, dishes, soup, porridge, and snacks (Table 2). Furthermore, TEI was also determined. The water content and energy for each food were calculated according to the Chinese Food Composition Table (6th edition) [17]. The primary reference level for TWI was derived from the AI values established by CNS for different age groups [9].

### 2.3. Statistical Analysis

Dietary data from the two- or four-day surveys were inputted into Excel sheets. Continuous variables with a normal distribution were expressed by their mean and standard deviation (SD). Those with a non-normal distribution were denoted by their median and interquartile range (IQR). Categorical variables were denoted by their frequency (*n*) and percentage (%). Mann–Whitney U test and Chi-Square test were employed to compare the differences in related variables between age groups. Moreover, age-adjusted partial correlation was utilized to assess the potential correlation. All data were calculated and analyzed via the SPSS software package version 26.0 (IBM, New York, NY, USA). A two-sided *p* value of <0.05 was considered as statistical significance.

## 3. Results

### 3.1. Age of the Study Participants

In summary, a total of 1360 eligible participants aged from 13 to 36 months (mean age: 24.4 ± 6.9 months) were included in the analysis. Among them, there were 670 boys (mean age: 24.4 ± 6.9 months) and 690 girls (mean age: 24.4 ± 6.9 months), accounting for 49.3% and 50.7%, respectively.

### 3.2. The Contribution of Water Intake from Different Sources

The data in Table 3 showed that the median daily TWI of the study population aged 13 to 36 months was 1079 mL. Of this, 670 mL (62.3%) came from beverages and 393 mL (37.7%) came from foods. In the water intake from beverages, plain water served as the main source, accounting for 300 mL (52.2%), followed by MMDs at 291 mL (45.6%), while the water intake from FVJs, SSDs, and PBDs was almost negligible, with a median of 0 mL. In terms of water intake from foods, staple food accounted for the highest contribution at 27.3%, followed by porridge (24.7%), dishes (22.4%), snacks (13.0%), and soup (12.6%).

Upon further analysis by age group, the older group exhibited a significant increase in TWI, the water intake coming from beverages, plain water, SSDs, PBDs, food, staple foods, dishes, soup, porridge, and snacks (*p* < 0.05), compared to the younger group; however, their water intake from MMDs significantly decreased from 336 mL to 263 mL (*p* < 0.05). The results with regard to the contribution of water intake from various sources revealed that, in the older group, the contribution of water from beverage to TWI, the contribution of water from MMDs to the water from beverages, and the contribution of water from staple food to the water from foods were significantly lower than those in the younger group (*p* < 0.05). Conversely, the water intake from beverages derived from plain water, SSDs, or PBDs, food-derived water, and soup-derived water all contributed considerably more water to TWI than did the younger group (*p* < 0.05).

### 3.3. Adherence to the AI Value: Comparisons with the TWI

**Figure 1:**
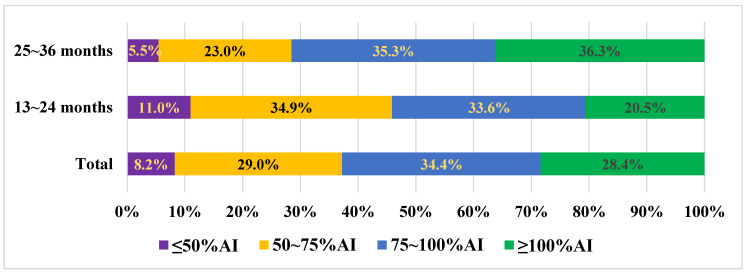
Percentages (%) of children aged 13~36 months according to compliance with AI value of TWI set by the Chinese Nutrition Society by age, segmented based on 50%AI, 75%AI, and 100%AI; AI: adequate intake, The chi-square test (χ^2^) was used to analyze the differences, yielding a chi-square value of 59.270 (*p* < 0.05), indicating a statistically significant difference between the two groups.

As shown in Figure 1, based on their TWI, the participants were divided into four groups compared with the AI value, <50%, 50~75%, 75~100%, and ≥100%, which corresponded to <650 mL, 650~975 mL, 975~1300 mL, and ≥1300 mL, respectively. Among the total population, only 28.4% of children had a TWI equal to or exceeding 1300 mL. After being categorized by age, the results indicated that the proportion of children with a TWI of 1300 mL or more and within the range of 975–1300 mL was notably higher in the older group compared to the younger group (36.3% vs. 20.5%, 35.3% vs. 33.6%), which were statistically significant (*p* < 0.05).

As depicted in Table 4, based on whether their actual TWI reached the AI of 1300 mL, children aged 13~24 months and 25~36 months were divided into two groups. Their daily TWI and contributions of water intake from different sources were also analyzed.

The median daily TWI among children aged 13~24 months whose daily TWI met the AI value (*n* = 140, group 1) was 1511 mL. This intake was primarily derived from beverages (989 mL, 63.9%) compared to foods (530 mL, 36.1%). For those who failed to meet the AI value (*n* = 542, group 2), the median daily TWI was much lower (926 mL) compared to group 1. The water intake from beverages (601 mL) and foods (306 mL) was significantly lower in group 2 compared to group 1, even though the water contributions to TWI from beverages (64.8%) and foods (35.2%) were similar between the two groups. When analyzing water from beverages, it was shown that the water intake from plain water and MMDs was significantly higher in group 1 compared to group 2 (500 mL vs. 265 mL and 408 mL vs. 307 mL, respectively) (*p* < 0.05). The water intake from staple foods, dishes, soup, porridge, and snacks in group 1 significantly surpassed those in group 2 in terms of water intake from foods (*p* < 0.01).

The median daily TWI of children aged 25–36 months was 1517 mL for those whose daily TWI achieved the AI value (*n* = 246, group 3). The contributions from food and beverages were 581 mL (39.0%) and 955 mL (61.0%), respectively. For those whose daily TWI did not reach the AI value (*n* = 432, group 4), the median daily TWI (1008 mL) was significantly lower than that of group 3. Although the contribution of water in beverages (59.5%) and food (40.5%) in group 4 was similar to that in group 3, the water intake from beverages (578 mL) and foods (386 mL) was significantly lower than those in group 3 (*p* < 0.05). Further analysis of the contribution of water intake from different sources revealed a trend similar to that of children aged 13~24 months.

### 3.4. Daily TEI from Different Sources

As detailed in Table 5, study participants had a median daily TEI of 762 kcal, with 272 kcal from beverages and 492 kcal from foods, representing 36.4% and 63.6% of TEI, respectively. In the energy intake from beverages, the median daily energy intake from MMDs was 260 kcal, making up 94.6% of the energy intake from beverages, while the energy intake from FVJs, SSDs, and PBDs was minimal, with a median of 0 kcal/day. In terms of daily energy intake from foods, staple food contributed the most calories with a total of 201 kcal (43.0%), followed by dishes (143 kcal, 33.4%), snacks (74 kcal, 18.3%), and porridge (8 kcal, 5.3%).

Further analysis by age group found that, compared with the younger group, the older group showed a significant increase in TEI, energy intake from food, staple foods, dishes, porridge, and snacks (*p* < 0.05) while experiencing a significant decrease in the energy intake from beverages and MMDs (*p* < 0.05). As for the contribution of energy intake, the results revealed that the older group had significantly lower contributions of energy from beverages to TEI and from MMDs to beverage energy than those in the younger group (*p* < 0.05), whereas the contribution of energy from SSDs or PBDs to the energy from beverages, energy from food to TEI, and the energy from dishes to energy from foods were significantly greater than those in the younger group (*p* < 0.05).

### 3.5. Partial Correlation Analysis

A partial correlation analysis was performed after controlling for age to investigate the correlations between various variables, including TWI, water and energy intake from beverages and different categories, water and energy intake from food and different categories, and TEI (Table 6). In terms of water intake, the TWI demonstrated a moderate correlation with water from beverages (r = 0.752), plain water (r = 0.634), and foods (r = 0.716). The water intake from beverages exhibited a strong correlation with plain water (r = 0.823), while the water intake from foods was moderately correlated with that from staple food, dishes, and porridge (r = 0.671, 0.694, and 0.654, respectively). In terms of energy intake, there was a robust correlation between TEI and energy from foods (r = 0.894), whereas TEI and energy from staple food and dishes had a moderate correlation (r = 0.621 and 0.708, respectively). We observed a robust correlation between energy from beverages and energy from MMDs (r = 0.959), while the energy from foods was moderately correlated with energy from staple food and dishes (r = 0.713 and 0.775, respectively). Regarding the correlation between energy and water intake, the TEI showed moderate correlations with water from foods and dishes (r = 0.657 and 0.644, respectively), while energy from foods showed moderate correlations with water from foods (r = 0.750) and dishes (r = 0.718).

## 4. Discussion

As far as current research is concerned, this represents the first-ever and an innovative attempt to look at the daily TWI and TEI as well as consumption patterns of toddlers (13–36 months) in China using a nationally representative sample. Encouragingly, the study revealed positive consumption trends, with plain water and MMDs being the primary contributors to water from beverages, and minimal consumption of SSDs. Nevertheless, a tremendous gap existed, as the water intake of most participants failed to meet the recommended AI value of water set by the CNS.

Before the study was conducted, the TWI of Chinese children aged 13~36 months remained unknown [9]. Until now, there have been only a few studies on the water intake of Chinese children, each with its own limitations. For instance, in Wang XJ et al.’s survey, despite having a large study sample of 5914, it only included children from four cities with higher economic levels, which was not representative of the general population [18]. Moreover, some studies focused only on measuring total fluid intake (TFI) rather than TWI, and have mistakenly equated these two [11,14,19]. During the toddler stage, both liquid and solid foods that provide energy, water, and other nutrients are crucial for meeting the child’s energy and nutrient needs [20,21]. As the study’s results showed, water intake from food in this age group did contribute to variations in TWI, and water intake from food should be calculated instead of being neglected. Therefore, our present survey could provide some valuable insights and strategies for subsequent investigations into TWI of children.

Currently, reports on toddlers’ water intake have been limited to only three countries. Of these studies, the ones conducted in France and Mexico were particularly noteworthy as they considered both water intake from food and beverages, whereas the study in the United States solely focused on beverage consumption [15,22,23]. Our research indicated that the median daily TWI of toddlers aged 13~24 months in China was 1015 mL, with 651 mL derived from beverages and 336 mL from foods, while that of toddlers aged 25~36 months was 1180 mL, with 696 mL coming from beverages and 449 mL from foods. This intake was slightly higher than the median daily TWI reported in France for different age groups: 999 mL (12~17 months), 985 mL (18~23 months), 991 mL (24~29 months), and 991 mL (30~35 months) [22], and lower than the mean daily TWI of toddlers aged 1–3 years reported in Mexico, which was 1283 mL, with 743 mL from beverages and 540 mL from foods [15]. These differences may be attributed to racial diversity, geographical location, cultural customs, economic level, dietary habits, feeding patterns, accessibility to beverages and food, and survey methodologies.

Plain water contributed differently to TWI, as we discovered when we looked more closely at the contributions of other sources. In our study, plain water contributed 30.3% to TWI for 13~24-month-olds, compared to 13% (12~17 months) and 19% (18~23 months) in France. For 25~36-month-olds, plain water contributed 34.6% in our study, versus 23% (24~29 months) and 25% (30~35 months) in France [22]. Additionally, our study determined that the median daily plain water intake among toddlers aged 13 to 36 months was 300 mL, whereas the average plain water intake in Mexico was 227 mL [15]. The intake of plain water and its contribution to TWI among toddlers in China are significantly higher compared to those in other regions. This difference could be explained by the widespread preference among Chinese individuals for drinking boiled water. This preference is not only a convention in China that is believed to aid digestion, improve circulation, and maintain internal balance, but also a common practice to eliminate pathogens from tap water, thereby enhancing its safety for drinking [24,25]. Despite the notably higher intake of plain water among Chinese toddlers, it did not translate into a significantly elevated water intake from beverages compared to toddlers in Mexico. This might be due to the higher intake of coffee, soda, and other beverages among Mexican toddlers, potentially influenced by the Mexican culture that often includes these beverages as part of their daily routine [15]. In our previous survey, we found that the daily consumption of soft drinks among preschoolers in China was 40 g/d [26]. However, in this study, we were pleased to see that Chinese toddlers consumed significantly fewer soft drinks, especially SSBs. The rising popularity of SSBs poses risks of obesity, dental issues, and other health concerns [26,27,28,29]. Therefore, it is advisable to avoid their consumption in early childhood.

In our study, Chinese toddlers showed poor compliance with the AI value (1300 mL/d) for TWI set by the CNS, with 71.6% of all ages failing to reach this value (79.5% for aged 13~24 months and 63.7% for aged 25~36 months). This is similar to the situation of 13~36-month-old toddlers in Mexico, with 62% of them not reaching the AI value (1300 mL/d) set by the Institute of Medicine in TWI [15]. However, in the survey conducted in France, nearly half of the toddlers aged 12~23 months had a TWI that reached the AI value (49% for those aged 12~17 months and 50% for those aged 18~23 months), whereas the proportion of toddlers aged 24~29 months and 30~35 months whose TWI did not reach the AI value was 88% and 84%, respectively. This may be due to the different AI recommendations set by EFSA (1100 mL/d for aged 12~23 months and 1300 mL/d for aged 24~35 months) [8,22]. The discrepancy in TWI recommendations among regions worldwide underscores the limitations of relying solely on TWI to assess hydration status. Introducing a gold-standard biomarker or other physical methods is deemed necessary, although some studies suggested that such approaches were still in their early stages [30].

Based on whether the TWI of toddlers reached the AI value set by the CNS, two age groups in our study showed similar trends in the intake of plain water and MMDs, which were the main contributors to water from beverages. For those with TWI reaching AI levels, their intake of plain water and MMDs was higher compared to those without TWI reaching AI levels. While parents often focus on offering complementary foods, maintaining a regular and adequate intake of water is important because toddlers have limited ability to perceive and express thirst, and they often encounter difficulties in self-feeding [30,31]. In addition to hydration, MMDs are considered to be good sources of calcium, phosphorus, magnesium, vitamin A, B vitamins, and vitamin D, making them ideal dietary components for improving the nutritional status of toddlers [26,32]. However, our research findings indicate that as children age, the consumption of MMDs significantly decreases. Especially for toddlers whose TWI did not reach the AI value, the median intake of MMDs in both age groups was lower than the recommended level of 350 g/d by the CNS [31]. These findings prompt us to reconsider strategies for enhancing dietary nutrition during early life stages and emphasize the importance of cultivating the habit of drinking MMDs and adequate water in toddlers under practical guidance.

There are some limitations to be acknowledged. First, the water demand of the human body is closely influenced by climate, temperature, and humidity [33]. Despite using a nationwide population sample, the collection of short-term water and dietary data cannot fully represent the annual food and water intake status of toddlers. Second, although a food atlas was used in this study, it should be noted that toddlers have relatively low levels of food and beverage intake each time, which may introduce bias into their estimation. Even with well-trained researchers conducting surveys, the amount of food or beverage provided by guardians to toddlers may differ from their actual intake, as adding supplementary food and learning to eat independently can lead to wastage or extra intake [30]. Third, toddlers are naturally active, and their physical activity levels have potential impacts on the water intake. However, the level of physical activity was not considered in this study. Finally, as a cross-sectional study, the relationship between water intake and children’s growth and development cannot be explored.

## 5. Conclusions

In summary, the results of this study suggest the need to pay attention to the quality of water intake among Chinese toddlers, which can be achieved by strengthening the education of guardians and implementing more scientific drinking water plans. In the future, broader coverage and more scientific data collection methods can be carried out to further verify the results of this study, and longitudinal follow-up can be conducted to explore the impact of water intake on children’s growth and development.

## Figures and Tables

**Table 1:** Classification of the beverage types.

Classification	Detailed Beverage Types
Plain water	Tap water, Bottled water (mineral water, purified water).
Milk and milk derivatives	Liquid milk (low fat milk, full fat milk), Powdered milk (whole or skimmed powdered milk), Yogurt, etc.
Fruit and vegetables juices	100% Fruit juice (bottled or freshly squeezed watermelon juice, pear juice, orange juice, etc.), 100% Fruit and vegetable juice (bottled or freshly squeezed cucumber and pineapple mixed juice, orange and carrot mixed juice, apple, and celery mixed juice, etc.).
Sugar-sweetened drinks	Carbonated beverages (Cola, Sprite, Fanta, etc.), Tea flavored beverage (bottled/boxed ice red tea, ice green tea, jasmine tea, etc.), Fruit flavored beverage (peach flavored, lemon flavored, grapefruit flavored, etc.), Milk flavored beverage (chocolate milk, banana milk, coconut milk, etc.), Functional beverages (probiotics, dietary fiber, vitamins, etc.), Milky tea (bubble tea, coconut jelly milk tea, fruit milk tea, etc.), etc.
Plant-based protein drinks	Soybean milk, almond juice, walnut juice, etc.

**Table 2:** Classification of food types.

Classification	Detailed Food Types
**Staple food**	
Cereals and cereals products	Rice, Steamed buns, Bread, Steamed twisted rolls, Fried dough sticks, Noodles, etc.
Compound processed products	Dumplings, Wonton, Hamburger, Sandwich, etc.
Others	Sweet potato, Starch vermicelli, etc.
**Dishes**	
Vegetables	Spinach, Cabbage, Skunk cabbage, Rape, Leaf lettuce, Amaranth, Chinese leek, Chrysanthemum, Garlic bolt, Cauliflower, Broccoli flower, Rice stem, Chinese cabbage, Carrot, Potato, Chinese yam, Taro, Bamboo shoot, Onion, Garlic, Lotus root, Cucumber, Summer squash, Towel gourd, Pumpkin, White gourd, Bitter gourd, Eggplant, Cowpea, Snap bean, Snow pea, Green soybean, Pea, Soybean sprout, Green gram sprouts, Mushroom, Laver, Sea frozen vegetables, Kelp seaweed, etc.
Soybeans and their products	Tofu, Shredded tofu, Dried bean curd, Tofu skin, etc.
Seafoods	River white fish, Grass carp, Catfish, Loach, Carp, Yellow croaker, Perch, Sea shrimp, River shrimp, Lobster, Sea crab, River crab, Oysters, Clams, Scallops, Cuttlefish, Octopus, etc.
Livestock meat	Pork, Pork (heart/liver/kidney/ear/blood/intestine), Trotters, Bacon, Spam, Beef, Beef tongue, Tripe, Mutton, Lamb liver, Lamb belly, etc.
Poultry	Chicken, Chicken (wings/legs/claw/liver, heart/gizzards/blood), Duck, Duck (wings/claw/tongue/intestine/liver/gizzard/blood), Goose, Goose (wings/foot/liver/gizzard/blood), Quail, Pigeon, etc.
Eggs	Hen’s egg, Duck egg, Goose egg, Quail egg, etc.
**Porridge**	Rice porridge, Millet porridge, Corn porridge, Black rice, and mixed bean porridge, etc.
**Soup**	Tomato egg soup, Bone soup, etc.
**Snacks**	
Fruits	Apple, Pear, Hawthorn, Peach, Apricot, Plum, Papaya, Cherry, Grape, Persimmon, Tangerines, Grapefruit, Watermelon, Honeydew melon, Strawberry, Kiwifruit, Mulberry, Banana, Mango, Litchi, Pitaya, Durian, etc.
Nuts	Walnut, Chestnut, Pine nut, Peanut, Sunflower seed, Pumpkin seed, watermelon seed, etc.
Others	Cake, Cookies, Chocolate, Ice cream, etc.

**Table 3:** Daily water intake (mL/day) and contribution of water intake from different sources (%) among children aged 13~36 months.

Variables	Total (*n* = 1360)		13~24 Months (*n* = 682)		25~36 Months (*n* = 678)		*Z*	*P*_1_	*Z*	*P*_2_
	P50 (P25, P75)	%	P50 (P25, P75)	%	P50 (P25, P75)	%				
Total water intake	1079 (886, 1336)	/	1015 (812, 1218)	/	1180 (949, 1428)	/	8.400	<0.001	/	/
Water from beverages	670 (516, 860)	62.3	651 (508, 834)	64.6	696 (527, 898)	60.1	2.927	0.003	−6.698	<0.001
Plain water	300 (200, 500)	52.2	300 (200, 400)	46.9	400 (250, 550)	57.6	7.768	<0.001	9.874	<0.001
MMDs	291 (200, 401)	45.6	336 (223, 430)	51.8	263 (183, 364)	39.6	−7.018	<0.001	−10.868	<0.001
FVJs	0 (0, 0)	0.6	0 (0, 0)	0.5	0 (0, 0)	0.6	0.999	0.318	0.935	0.350
SSDs	0 (0, 0)	0.8	0 (0, 0)	0.5	0 (0, 0)	1.1	5.651	<0.001	5.593	<0.001
PBDs	0 (0, 0)	0.8	0 (0, 0)	0.3	0 (0, 0)	1.1	6.471	<0.001	6.444	<0.001
Water from foods	393 (270, 529)	37.7	336 (229, 463)	35.4	449 (319, 589)	39.9	10.306	<0.001	6.698	<0.001
Staple food	84 (52, 138)	27.3	78 (44, 133)	29.3	91 (60, 144)	25.4	4.561	<0.001	−2.805	0.005
Dishes	77 (41, 129)	22.4	65 (32, 110)	22.0	90 (52, 138)	22.8	7.321	<0.001	1.751	0.080
Soup	38 (1, 80)	12.6	25 (0, 65)	11.6	50 (15, 95)	13.5	7.321	<0.001	4.395	<0.001
Porridge	76 (22, 160)	24.7	59 (16, 120)	23.6	95 (26, 192)	25.8	5.647	<0.001	1.848	0.065
Snacks	39 (18, 74)	13.0	36 (15, 95)	13.5	42 (20, 81)	12.5	3.569	<0.001	−1.480	0.139

MMDs: Milk and milk derivatives; FVJs: fruit and vegetable juices; SSDs: sugar-sweetened drinks; PBDs: plant-based protein drinks. *P*_1_ was for the differences in the daily water intake (mL/day) between two age groups through statistical comparisons. *P*_2_ was for the differences in the contribution of water intake from different sources to TWI, water from beverages and water from foods (%) between two age groups through statistical comparisons.

**Table 4:** Daily water intake (mL/day) and contribution of water intake from different sources (%) among children aged 13~36 months categorized by AI values of TWI set by the Chinese Nutrition society.

Variables	13~24 Months (*n* = 682)								25~36 Months (*n* = 678)							
	≥AI (*n* = 140), Group 1		<AI (*n* = 542), Group 2		*Z*	*P*_1_	*Z*	*P*_2_	≥AI (*n* = 246), Group 3		<AI (*n* = 432), Group 4		*Z*	*P*_1_	*Z*	*P*_2_
	P50 (P25, P75)	%	P50 (P25, P75)	%					P50 (P25, P75)	%	P50 (P25, P75)	%				
Total water intake	1511 (1366, 1689)	/	926 (759, 1073)	/	−18.256	<0.001	/	/	1517 (1401, 1712)	/	1008 (835, 1164)	/	−21.669	<0.001	/	/
Water from beverages	989 (831, 1186)	63.9	601 (475, 714)	64.8	−14.486	<0.001	0.839	0.401	955 (774, 1136)	61.0	578 (459, 724)	59.5	−16.968	<0.001	−1.154	0.248
Plain water	500 (400, 800)	53.9	265 (150, 400)	45.1	−12.155	<0.001	−5.387	<0.001	600 (500, 800)	62.4	300 (200, 500)	54.8	−14.716	<0.001	−5.192	<0.001
MMDs	408 (340, 526)	44.4	307 (210, 405)	53.6	−7.635	<0.001	5.400	<0.001	301 (221, 421)	34.7	241 (172, 337)	42.3	−5.932	<0.001	5.315	<0.001
FVJs	0 (0, 0)	0.7	0 (0, 0)	0.5	−2.661	0.008	−2.391	0.017	0 (0, 0)	0.7	0 (0, 0)	0.6	−3.037	0.002	−2.626	0.009
SSDs	0 (0, 0)	0.5	0 (0, 0)	0.5	0.014	0.989	0.122	0.903	0 (0, 0)	1.2	0 (0, 0)	1.0	−2.659	0.008	−2.107	0.035
PBDs	0 (0, 0)	0.5	0 (0, 0)	0.3	−0.586	0.558	−0.522	0.602	0 (0, 0)	1.0	0 (0, 0)	1.3	−0.121	0.904	0.341	0.733
Water from foods	530 (400, 669)	36.1	306 (213, 404)	35.2	−11.649	<0.001	−0.839	0.401	581 (470, 741)	39.0	386 (276, 490)	40.5	−12.496	<0.001	1.154	0.248
Staple food	121 (75, 231)	30.5	69 (39, 113)	29.1	−7.716	<0.001	−0.443	0.658	112 (70, 178)	23.9	83 (55, 126)	26.3	−5.497	<0.001	2.963	0.003
Dishes	94 (56, 156)	21.3	56 (28, 101)	22.3	−6.123	<0.001	0.673	0.501	118 (63, 186)	21.4	79 (49, 125)	23.7	−5.709	<0.001	2.326	0.020
Soup	58 (0, 100)	13.4	20 (0, 58)	11.0	−4.886	<0.001	−1.948	0.051	75 (31, 138)	14.4	38 (10, 77)	13.1	−6.143	<0.001	−1.957	0.050
Porridge	85 (27, 179)	21.3	56 (16, 114)	24.1	−3.283	0.001	1.231	0.218	160 (53, 289)	27.8	80 (17, 152)	24.4	−6.103	<0.001	−2.037	0.042
Snacks	57 (28, 95)	13.5	31 (14, 59)	13.5	−5.613	<0.001	0.176	0.861	59 (27, 103)	12.5	35 (19, 67)	12.5	−5.730	<0.001	0.107	0.915

MMDs: Milk and milk derivatives; FVJs: fruit and vegetable juices; SSDs: sugar-sweetened drinks; PBDs: plant-based protein drinks. *P*_1_ was for the differences in the daily water intake (mL/day) between two age groups through statistical comparisons. *P*_2_ was for the differences in the contribution of water intake from different sources to TWI, water from beverages and water from foods (%) between two age groups through statistical comparisons.

**Table 5:** Daily energy intake (kcal/day) and contribution of energy intake from different sources (%) among children aged 13~36 months.

Variables	Total (*n* = 1360)		13~24 Months (*n* = 682)		25~36 Months (*n* = 678)		*Z*	*P*_1_	*Z*	*P*_2_
	P50 (P25, P75)	%	P50 (P25, P75)	%	P50 (P25, P75)	%				
Total energy intake	762 (589, 990)	/	719 (559, 923)	/	811 (622, 1035)	/	5.250	<0.001	/	/
Energy from beverages	272 (185, 367)	36.4	285 (207, 373)	40.8	248 (164, 356)	32.0	−4.694	<0.001	−9.390	<0.001
MMDs	260 (174, 349)	94.6	279 (195, 364)	96.6	229 (154, 336)	92.6	−5.285	<0.001	−5.218	<0.001
FVJs	0 (0, 0)	1.7	0 (0, 0)	1.4	0 (0, 0)	2.0	0.840	0.401	1.076	0.282
SSDs	0 (0, 0)	2.0	0 (0, 0)	1.2	0 (0, 0)	2.8	6.127	<0.001	6.302	<0.001
PBDs	0 (0, 0)	1.7	0 (0, 0)	0.8	0 (0, 0)	2.6	6.442	<0.001	6.612	<0.001
Energy from foods	492 (331, 684)	63.6	422 (280, 611)	59.2	535 (388, 740)	68.0	8.526	<0.001	9.390	<0.001
Staple food	201 (115, 311)	43.0	179 (100, 284)	43.6	223 (144, 336)	42.4	6.199	<0.001	−1.171	0.242
Dishes	143 (94, 216)	33.4	119 (70, 187)	31.9	167 (121, 244)	34.8	10.680	<0.001	4.488	<0.001
Porridge	8 (2, 30)	5.3	6 (2, 25)	5.6	11 (3, 32)	5.1	3.422	0.001	1.135	0.256
Snacks	74 (37, 135)	18.3	67 (32, 127)	18.9	81 (41, 142)	17.7	3.364	0.001	−1.194	0.232

MMDs: Milk and milk derivatives; FVJs: fruit and vegetable juices; SSDs: sugar-sweetened drinks; PBDs: plant-based protein drinks. *P*_1_ was for the differences via statistical comparisons between age groups in the daily energy intake (kcal/day) of different sources; *P*_2_ was for the differences via statistical comparisons between age groups in the contribution of energy intake from different sources to total energy intake/energy from beverages/energy from foods (%).

**Table 6:** Partial correlation through various variables, including TWI, water and energy intake from beverages and their different sources, water and energy intake from foods and their different sources, and TEI (adjusted for age).

Variables	Total Water Intake	Water from Beverages	Water from Foods	Total Energy Intake	Energy from Beverages	Energy from Foods
Total water intake	1.000	0.752 ***	0.716 ***	0.562 ***	0.325 ***	0.490 ***
Water from beverages	0.752 ***	1.000	0.077 **	0.182 ***	0.421 ***	−0.009
Water from plain water	0.634 ***	0.823 ***	0.087 **	0.011	0.024	0.000
Water from MMDs	0.382 ***	0.595 ***	−0.052	0.249 ***	0.681 ***	−0.073 **
Water from FVJs	0.308 ***	0.114 ***	0.345 ***	0.295 ***	0.073 **	0.309 ***
Water from SSDs	0.066 *	0.067 *	0.029	0.116 ***	0.133 ***	0.067 *
Water from PBDs	0.249 ***	0.044	0.331 ***	0.170 ***	−0.068 *	0.236 ***
Water from foods	0.716 ***	0.077 **	1.000	0.657 ***	0.045	0.750 ***
Water from staple food	0.513 ***	0.099 ***	0.671 ***	0.510 ***	0.046	0.576 ***
Water from dishes	0.472 ***	0.019	0.694 ***	0.644 ***	0.075 **	0.718 ***
Water from soup	0.295 ***	0.119 ***	0.320 ***	0.129 ***	0.086 **	0.106 ***
Water from porridge	0.421 ***	−0.017	0.654 ***	0.227 ***	−0.076 **	0.307 ***
Water from snacks	0.377 ***	0.048	0.519 ***	0.508 ***	0.100 ***	0.545 ***
Total energy intake	0.562 ***	0.182 ***	0.657 ***	1.000	0.534 ***	0.894 ***
Energy from beverages	0.325 ***	0.421 ***	0.045	0.534 ***	1.000	0.099 ***
Energy from MMDs	0.313 ***	0.438 ***	0.009	0.461 ***	0.959 ***	0.036
Energy from FVJs	0.217 ***	0.101 ***	0.221 ***	0.233 ***	0.087 **	0.228 ***
Energy from SSDs	0.040	0.048	0.010	0.124 ***	0.216 ***	0.032
Energy from PBDs	0.243 ***	0.037	0.328 ***	0.173 ***	−0.066 *	0.239 ***
Energy from foods	0.490 ***	−0.009	0.750 ***	0.894 ***	0.099 ***	1.000
Energy from staple food	0.367 ***	0.057 *	0.494 ***	0.621 ***	0.034	0.713 ***
Energy from dishes	0.353 ***	−0.062 *	0.599 ***	0.708 ***	0.112 ***	0.775 ***
Energy from porridge	0.354 ***	−0.018	0.554 ***	0.293 ***	−0.056*	0.374 ***
Energy from snacks	0.256 ***	0.05	0.009	0.378 ***	0.587 ***	0.109 ***

MMDs: Milk and milk derivatives; FVJs: fruit and vegetable juices; SSDs: sugar-sweetened drinks; PBDs: plant-based protein drinks. * *p* < 0.05, ** *p* < 0.01, *** *p* < 0.001.

## Data Availability

The data utilized in this study are available upon request from the corresponding author.

## Abbreviations

TWI	Total water intake
AI	Adequate intake
TEI	Total energy intake
MMDs	Milk and milk derivatives
FVJs	Fruit and vegetables juices
SSDs	Sugar sweetened drinks
PBDs	Plant-based protein drinks
SD	Standard deviation
IQR	Median and interquartile range
CNS	China Nutrition Society
DSIYC	The cross-sectional dietary intake survey of infants and young children
